# New challenges in modern vaccinology

**DOI:** 10.1186/s12865-015-0075-2

**Published:** 2015-03-26

**Authors:** Mireille Centlivre, Béhazine Combadière

**Affiliations:** Sorbonne Universités, UPMC University Paris 06, UMR_S CR7, Centre d’Immunologie et des Maladies Infectieuses- Paris, F-75013 Paris, France; Centre d’Immunologie et des Maladies Infectieuses CIMI-Paris, 91 Boulevard de l’Hôpital, 75013 Paris, France

**Keywords:** Vaccination, Humanized mouse model, Correlates of protection, Pathogens, Immune response, Route of administration, Adjuvants

## Abstract

Vaccination has been a major advance for health care, allowing eradication or reduction of incidence and mortality of various infectious diseases. However, there are major pathogens, such as Human Immunodeficiency Virus (HIV) or the causative agent of malaria, for which classical vaccination approaches have failed, therefore requiring new vaccination strategies. The development of new vaccine strategies relies on the ability to identify the challenges posed by these pathogens. Understanding the pathogenesis and correlates of protection for these diseases, our ability to accurately direct immune responses and to vaccinate specific populations are such examples of these roadblocks. In this respect, the use of a robust, cost-effective and predictive animal model that recapitulates features of both human infection and vaccination is currently a much-needed tool. We discuss here the major limitations faced by modern vaccinology and notably, the development of humanized mice for assessing the immune system, along with their potential as vaccine models.

## Introduction

One of the major advances in biomedical sciences resides in vaccination, which has allowed the eradication or the reduction of incidence and mortality of various infectious diseases [1]. Smallpox eradication is one of the best examples of vaccine efficacy.

Various types of vaccines have been developed and applied in humans, and can be classified into two main groups [2]. The first type of vaccine consists of live-attenuated pathogens, which have been used successfully against diseases such as smallpox, measles, polio and yellow fever. These vaccines mimic natural infection, but in a weakened non-pathogenic fashion. The second group comprises a wide range of vaccines, including inactivated toxins (diphtheria, tetanus), subunit preparations (hepatitis B), carbohydrate cocktails (pneumococcus) and conjugate vaccines (meningococcus, haemophilus influenza type B). In contrast with live-attenuated vaccines that confer lifelong memory, the second vaccination group usually requires adjuvants to enhance the induced immune response as well as boosting strategies that maintain protective immunity.

Despite this major step ahead for global public health and obvious benefits over the past century, vaccination faces new challenges amidst a world of quickly evolving pathogens. Specifically, classical vaccination approaches for many pathogens have failed because the capacity to generate fundamental knowledge regarding the pathogenesis of these infectious diseases and the ability to determine correlates of protection progress at a slower pace. Further, the capacity to direct the types of immune responses needed to confer protection through vaccination and to protect specific patient groups are affected by the relevance and/or lack of adequate animal models.

### Major infectious diseases for which no vaccine exists

For a wide range of pathogens, classical vaccination approaches have achieved limited success. All of these pathogens failed in the vaccine development path for different reasons, which are addressed here.

1. High antigenic variability and immune evasion. Some viruses such as Human Immunodeficiency Virus (HIV) and Hepatitis C Virus (HCV) are characterized by a high antigenic variability [3]. Their high mutation rate allows them to evade immune responses by modifying their target immunogens during the course of infection. In addition to the high intraspecies variability, several subtypes of these pathogens co-exist, adding a layer of complexity to developing broadly effective vaccines. Such immune evasion is also a major problem for the development of a universal Influenza vaccine, where annual updating of the viral strains and targets in vaccines is required for seasonal vaccination. Similarly, malaria represents a complex hurdle for vaccination [4]. At each step of the parasite’s complex life cycle, its morphology and expressed antigens change. When combined with allelic polymorphisms, these mutations allow the parasite to evade the host immune response.
2. Disease enhancement. Dengue virus (DENV) comprises 4 serotypes. After dengue infection, neutralizing antibodies are generated, conferring life long immunity against the infecting serotype. However, cross-reactive antibodies against other non-infecting serotypes are also generated and believed to increase the severity of subsequent infections by other dengue serotypes through antibody-dependent enhancement [5]. One of the challenges facing the development of a vaccine against DENV will be to induce a protective antibody response against all four DENV serotypes. In addition, the protective immune responses should be durable and equally effective against all 4 dengue serotypes to avoid an incomplete immune response, which would further facilitate and enhance pathogenesis. The recent results of the phase IIb Sanofi tetravalent DENV vaccine highlighted this difficulty to induce such equally protective immunity against the 4 serotypes [6]. This phenomenon was also observed for respiratory syncytial virus (RSV) in a human vaccination setting, where an incomplete immune response after vaccination lead to vaccine-mediated disease enhancement [7].
3. Time of the infection. RSV is one of the main causes of respiratory infection in infants and efficient vaccine against RSV represents an important yet unmet medical need. A major issue with RSV is its timing of infection, where infants in their first 6 months are at highest risk of severe RSV disease, during a period where the immune system is still immature [7]. Inducing effective immune responses that will last in newborns is particularly challenging and may require maternal immunization strategies with mother-to-fetus transmission of protective antibodies.
4. Neglected tropical diseases. Viruses that are circulating in tropical regions and cause hemorrhagic fever, face a lack of interest in terms of an investment in vaccine development, which does not necessarily reflect an inability to elicit effective vaccine-induced immune responses [8]. Indeed, for Junin virus (New World Arenavirus), a live-attenuated vaccine is used in Argentina, but this local vaccine has not been approved for use in other countries. Similarly, for the hemorrhagic fever with renal syndrome (HFRS) caused by Old World Hantaviruses, a local vaccine used in Korea and China have reduced the number of HFRS cases since its implementation.

### Deciphering correlates of protection

Whereas most successful vaccines have been developed empirically, there is now a need for understanding the pathogenesis of the infecting organism as well as the disease-specific mechanisms of protective immunity and immune evasion [2,9]. Most of the pathogens for which effective vaccines exist are characterized by a primary infection, which results in long-lasting resistance in the surviving host. As a consequence, vaccines were developed to induce an immune response that mimics the natural infection. Some pathogens that cause persistent infection and also promote the development of cancer, such as Hepatitis B and Papilloma viruses, can now be prevented by vaccines that deliver virus-like particles.

The induction by vaccines of antibodies that confer sterilizing protection against pathogens is usually defined in vaccination as the correlates of protection. However, for many diseases, we do not know which arms of the immune system are responsible for conferring protection, e.g. humoral versus cellular immunity, whether systemic or mucosal immunity should be induced for sterilizing protection. Parameters for the maintenance of protective immunity over years also have yet to be elucidated. In addition, for pathogens that do not induce robust resistance after primary natural infection, it is unclear how to confer sterilizing protection through vaccination. Systems biology approaches are one of the favored strategies used to decipher correlates of protection and predict vaccine efficacy [10-12]. Systems biology is a combination of omic technologies and computational tools that can be used to obtain quantitative, qualitative and integrated analyses at genomic, proteomic and cellular levels. This multiparametric approach helps define the innate signatures that are induced early after infection and/or vaccination and the subsequent adaptive response in humans, and as such gives a global picture of the complex interaction between the innate and adaptive arms of the immune system in one individual at a certain time. This has already been applied in the context of vaccinology studies against yellow fever and influenza [9].

Systems biology is a powerful tool to measure functional signatures of T cell and B cell responses and may shift the vaccinology dogma from correlates of protection as a single parameter important for vaccine efficacy to co-correlates of protection that combine multiple variables. In addition, not only the type of immune responses needed to confer protection has to be deciphered (correlates or co-correlates of protection), but also these correlates and co-correlates of protection have to be defined in particular populations, including newborn, infants, teenagers, adults, and the elderly; immunodeficient, pregnant individuals and individuals with autoimmune disease. Moreover, antigen design for avoiding immune escape is of importance, implying that balance between protective and enhancing epitopes should be defined as well as the mechanisms leading to the immunodominance of the irrelevant epitopes. The fields of antigen design benefits now from both reverse vaccinology and structural vaccinology. Reverse vaccinology, based on the sequencing of the genomes of pathogens, allows the *in silico* determination of putative candidate vaccine antigens that were not found by traditional methods. The recognized success of reverse vaccinology has been shown in obtaining an effective licensed vaccine against meningococcus type B [13]. Structural vaccinology, based on information of the 3D structure of the HIV envelope protein, is another key component that may lead towards development of successful vaccines against this virus or similarly, RSV [14,15].

### How to shape the adaptive immune response

One of the main challenges in vaccination, knowing or not correlates of protection, is to direct the immune system toward responses that would confer protection. How can potent antibody response be induced? How can Th1/Th2/Th17 responses be balanced? How can mucosal immunity be induced? How can long-lasting memory cells be induced?

For example, potent broadly neutralizing antibodies against HIV proteins have been discovered but all of these antibodies exhibit a particularly high level of hypersomatic mutations [14], further complicating the task of generating high affinity antibodies through vaccination. What is clear is that innate immunity has a central role in programming the adaptive immune response and consequently the protective one. Manipulating innate immunity at different levels, as presented below, may thus impact the outcome of protection by vaccination.

1. Adjuvants. Adjuvants have multiple facets. They are used in non live-attenuated vaccines in order to improve vaccine efficacy through increased antibodies titers, CD4 T cell frequencies and/or enhanced duration of the vaccine-induced immune responses [16]. They may influence the isotype class switching of antibodies and modulate the Th balance responses (Th1/Th2/Th17). In terms of vaccine manufacturing and large-scale production, use of adjuvants permits a reduction in the antigen dose and the number of doses required to provide protection. Few adjuvants are currently used in licensed vaccines, which are mainly added to enhance humoral immunity. However, a battery of new adjuvants is under preclinical or clinical development and testing [16]. Identifying their precise mechanisms of action will allow us to gain additional information about safety and insight on how to shape the nature of immune responses and duration of memory responses. In addition, adjuvants may be combined in the same vaccine formulation to maximize immunogenicity.
2. Vectors. Numerous vectors, replication competent or incompetent, have been developed for vaccination and characterized in preclinical models and clinical trials [17,18]. They are able to induce cytotoxic T cell responses in addition to antibody responses. Depending on the infectious agent, one vector will be preferred to another according to nature of immune responses necessary for protection. However, one major constraint to overcome is the potential pre-existing immunity to some vectors, which may limit the induction of the desired immune response against a pathogen or even favor the replication of the pathogen targeted by the vaccine vector. This has been observed in the Merck STEP trial, where pre-existing immunity against the vector, a recombinant Adenovirus type 5, led to an increased incidence of HIV infection [19].
3. Route of vaccine administration. Intramuscular and subcutaneous vaccination routes are the main administration modes. However, antigen-presenting cells (APC) are poorly represented in the muscle and direct priming of T cells is impaired, as myocytes lack expression of major histocompatibility complex (MHC) class II and costimulatory molecules. Consequently, adjuvants are necessary to enhance APC activation and infiltration into and around the intramuscular vaccination site. In addition, these routes of immunization favor systemic immunity and not mucosal tissues-associated immune responses. This is of importance when the pathogen’s portal of entry is the mucosal tissue.
  New strategies are being developed for an alternative mode of administration via mucosal tissues (intranasal, oral, sublinguinal, intrarectal and intravaginal) or cutaneous tissues (intradermal, transcutaneous, percutaneous). We have a special interest in the skin [20], since there is a higher density of APC is present in the skin epidermis (Langerhans cells) and dermis (dendritic cells). As APC are key players in the induction and shaping of immune responses, it is therefore tempting to use skin as a target organ for vaccination. Cutaneous vaccine immunization results in better antigen distribution and sustained APC recruitment into draining lymph nodes as compared to intramuscular administration. Consequently, the features of the generated immune response differ according to the administration route [21,22]. In addition, many studies in humans have demonstrated that intradermal (ID) vaccination induced immunogenicity similar to intramuscular administration but with smaller antigenic doses [20]. However, at similar doses, a superior immunogenicity was observed in the elderly population after administration of an ID influenza vaccine [23].

### Specific population groups to vaccinate

Improved health care, with reduction of infant mortality and decreased mortality in older age, leads to increased life expectancy. This impacts vaccination campaigns, as new target groups [24] such as the elderly population, who are more prone to develop infectious diseases, should be taken into account for new effective vaccination strategies. Indeed, senescence of the immune system in the elderly makes them more vulnerable to infections but also renders them less responsive to vaccination. Specific strategies to amplify the immune response, probably through combination of adjuvants, may be required. This should also take into account malnutrition and obesity, which may change outcomes of vaccination. Furthermore, there is more and more evidence of the impact of the microbiome on immunity and consequently on responses to vaccines.

In other specific groups of patients such as immunodeficient people or pregnant women, live-attenuated vaccines have to be avoided and switched toward the second group of vaccines, comprising inactivated, subunit, carbohydrates or conjugated vaccines. In addition, for some pathogens such as RSV, where protection in newborns is required shortly after birth, maternal immunization strategies have to be developed to compensate for the immaturity of the newborn immune system and the difficulty to generate potent immune responses at an early age.

Last, opponents of vaccination represent a growing group in developed countries. For example, measles outbreaks are more frequently observed; notably one case was documented in the USA that started from an unvaccinated child in an undervaccinated population [25]. Strategies should be undertaken to improve the social acceptability of vaccines.

### Which animal models should be used for preclinical vaccine development?

Preclinical vaccine development is limited by the lack of adequate animal models. Indeed, whereas mice have enormously contributed in understanding the immune system ontogeny and function, these animal models exhibit limitations for human vaccine development. Notably, the dose of antigen and adjuvants delivered are not representative of the corresponding human dose; the route of antigen administration varies between mice and humans as well as the expression of pattern recognition receptors important for adjuvant efficacy. In addition, no protection experiments can be performed with wild-type pathogens when dealing with strictly human-tropic pathogens, such as HIV and DENV.

Non-human primates and in particular, chimpanzees are another currently used preclinical model that have been essential for the development of several vaccines, including the hepatitis B one, but their elevated costs as well as their restriction of utilization, which are often due to ethical reasons, limits their large use. In addition, HIV vaccination studies have shown limitations of such models as different results were obtained in humans as compared to the preclinical assessment in monkeys. Indeed, SIV sequences are not as diverse as HIV sequences; restriction factors such as TRIM5α or the (absence of the) prevalence of some vaccine vectors in monkeys, such as adenoviruses, may impact the outcomes of the vaccine studies.

To overcome these limitations, efforts have been undertaken to generate adequate predictive animal preclinical models, easy to generate, cost-effective and allowing an *in vivo* approach of the human immune system. Such models would allow recapitulation of the characteristics of infection by human pathogens and modeling vaccination studies, consequently accelerating the transfer of vaccines as well as new therapeutics from preclinical to clinical stages.

The search for such models has intensified, resulting in the construction of mice humanized for the immune system (Human Immune System or HIS mice). These models mainly arise from the xenotransplantation of human hematopoietic cells and/or tissues, allowing the long-term establishment of components of human immunity in permissive immunodeficient mice. The generation of new immunodeficient mouse strains – in particular NOD/SCID/IL2Rγ_c_^-/-^ (NSG/NOG) and BALB/c Rag-/-IL2Rγ_c_^-/-^ (BRG) - has led to considerable improvements for acceptance of human xenofgrafts [26-30]. A single injection of human hematopoietic stem/progenitor cells allows the development, maturation and long-term maintenance of a multi-lineage human immune system, with all the major human hematopoietic cell populations found in the reconstituted HIS mice [26-30].

Interestingly, these HIS mice can be infected by human-specific lymphotropic pathogens targeting cells of the immune system such as HIV and DENV [31]. HIS mice are attractive tools for investigating pathogenesis of some infectious diseases in a human setting, as well as new live-attenuated HIV vaccines [32]. Several therapies have already been preclinically tested in these animal models and are mainly against HIV, such as microbicides or gene therapy [33,34]. However, induction of strong humoral and cellular immune responses in HIS mice still represents a major challenge. Human B and T cell responses remain suboptimal in HIS mice after immunization and are mostly detectable in infectious settings. Analysis of the antigen-specific B cell repertoire at the clonal level after commercial vaccine inoculation shows mainly an IgM response with a restricted level of somatic hypermutations [35]. Human T cells generated in NSG-HIS mice are educated in the context of a murine thymus, thus restricted to mouse MHC molecules. After immunization of NSG-HIS mice, proper CD4^+^ and CD8^+^ T-cell interactions with human APC may therefore be impaired, which may in turn limit B cell responses and the establishment of an IgG antibody response [36]. HIS mice transgenic for human MHC (HLA) class I and/or II molecules have therefore been generated and exhibit improved T cell functionality and improved IgG responses [37-39]. Nevertheless, further improvements are required to obtain strong adaptive immune responses. One potential strategy is to improve the density and/or functionality of human APC that are underrepresented in HIS mice. Supplementation of HIS mice with human cytokines implicated in the development and/or maturation of such cell compartments has demonstrated incremental optimization, further moving humanized mice towards valuable preclinical vaccine models [40,41]. Some vaccine strategies can already be tested in HIS mice, such as targeted vaccine delivery of Epstein-Barr virus nuclear antigen 1 to DC via the DEC205 receptor [42] or DC immunotherapy [43]. Although HIS mice may require some optimization for universal vaccine development and delivery, they already offer several possibilities for obtaining crucial information about the pathogenesis of some infections or the modulation of innate immunity by adjuvants.

## Conclusions

Despite major advances in health care made through massive vaccine campaigns over the past century, the field of vaccination faces new challenges. However, identifying these challenges is already one big step. In addition, technological advances in vaccine discovery, reverse and structural vaccinology, systems biology and immune monitoring, together with the optimization of preclinical animal models such as HIS mice, should help us bridge the gap to designing a new range of vaccines against the causative agents of current infectious diseases.

## Abbreviations

APC: Antigen-presenting cells
DENV: Dengue virus
HCV: Hepatitis C Virus
HFRS: Hemorrhagic fever with renal syndrome
HIS: Human Immune System
HIV: Human immunodeficiency virus
ID: Intradermal
MHC: Major histocompatibility complex
RSV: Respiratory syncytial virus
